# Notes and News

**Published:** 1891-08-15

**Authors:** 


### NOTES AND NEWS.

COLLECTED AND COMMUNICATED.

The Local Government have called upon the Guardians of the Kettering Union to show cause why a writ of *mandamus* should not issue commanding them to appoint a vaccination officer for the Kettering sub-district of the Kettering Union. A vacancy occurred in September, 1890, and the Guardians refused to appoint a successor, the last refusal being in July, 1891, and the Local Government Board, considering the matter was most important, were desirous of compelling the Guardians to make the appointment.—Their lordships granted a rule *nisi*, and in view of the urgency made the rule returnable during the vacation.

At the quarterly court of Governors of the Hospital for Consumption, Brompton, on the 6th ult. the Secretary (Mr. Dobbin) stated that since the annual court the whole of the 321 beds in the two buildings had remained occupied until quite recently, when the admissions to the older hospital were stopped, it being necessary to close the wards for the periodical cleaning, painting, &c. In addition to the treatment of patients in the hospital, 98 had been sent to the convalescent home at Sandgate, at the expense of the hospital, in order to confirm the improvement which they had received at Brompton. The Committee desired to remind the public that this is the Jubilee year of the hospital, which, it is hoped, may be marked by specially generous contributions. To those who preferred an object more definite for their liberality, the fund for the needful enlargement of the chapel may be commended to their notice. The following bequests had been announced:—Rev. C. R. B. Chetwynd, £100; Mr. James Smith, £1,000 duty free; Miss C. Graham, £636 1s. 10d. stock; Rev. T. L. Echalaz, £500, duty free. The number of patients admitted since May 28th, was 317; discharged, many greatly benefited, 283; died, 47; new out-patient cases, 2,314.

So many are the orphanages of to-day that we must almost wonder if any fatherless little ones can be left unprovided with a shelter. But there is a class which needs a home quite as much as those who have been deprived by death of father and mother—children either actually abandoned by their relations, or practically so. To these a refuge such as St. Augustine's Home for Boys, at Clewer, is indeed a Godsend. It possesses the advantage, from a home point of view, of being carried out on a small scale, only fifty boys being admitted. The disadvantage of small numbers, from an economical point of view, seems to have been overcome in the case of St. Augustine's Home, the boys being maintained and educated at the moderate cost per head of £24. The lads are well clad, and there is no stinting in the food. They are given employment as early as possible, when such is procurable in the neighbourhood, the boys treating St. Augustine's as their home until they are ready for a more independent life. There is a branch of the Home at Middlestown, in Yorkshire, where some of the elder boys do excellent colliery work. Everything is done by those in charge to develop an independent, self-respecting character, and the work, which is dependent on voluntary contributions, is well worthy of support.

At the annual meeting of the Trustees of the Manchester Royal Infirmary on the 30th ult., it was decided to make new regulations on the subject of students' fees at the Infirmary. The sums derived from this source have been steadily increasing, and amounted to upwards of £3,600 during the past year. The proceeds have hitherto been mainly devoted to defraying the cost of tuition, but in future a certain percentage of them will be paid to the managers of the Infirmary.

A great deal of opinion has been expressed from without upon the subject of the proposed hospital extension for Manchester, but we have heard very little from within the walls of the Infirmary. That this reticence has not been unintentional was made pretty clear at the annual meeting of the Trustees of the Institution. The Board apparently intend to choose their own time and method of stating their line of procedure. One thing, however, may be expected, and that is that the Infirmary authorities will not let the new buildings occupy any ground without the confines of the present Institution without a struggle.

A little knowledge is stated to be a dangerous thing. A sufficient acquaintance with the properties of testing fluids to enable a patient of the humbler classes to select a particular poison by means of which to put an end to her life shows that increasing knowledge may prove more dangerous still. Ellen Gertch, the wife of a Swiss waiter, and a patient at the Temperance Hospital, Hampstead Road, in the absence of the nurse from the ward, took a bottle of sulphuric acid from the row of testing fluids, and drank a sufficient quantity to cause her death.

At the last meetings in connection with the visit of the British Medical Association to Bournemouth, in the public medicine sections, a discussion was initiated by Sir Spencer Wells on the burial of the dead, and the general opinion was favourable to cremation. An adjourned discussion on hypnotism took place in the physiological section, and resulted in the passing of a resolution setting forth the desirability of calling upon the Government to prohibit the exhibition of hypnotism and hypnotic subjects.

The Deptford Vestry have received a long report from a special committee appointed to inquire into the origin, objects, and mode of distribution of the parish charities. The report states that during the last 300 or 400 years forty-seven charitable funds had been given for the benefit of the poor of Deptford. The first, dated 1563, amounted to 12s. yearly for "bread, beef, and beer for twelve parishioners on December 23," had lapsed. An unknown donor left a sum of money for "pea haulm and rushes for the parish church," and bread for distribution on Good Friday. The value of this is now £2 4s. 10d. per annum, which is expended in bread and coal for the poor at Christmas. Bequests, dated 1640, 1673, 1678, and 1692, have lapsed. Colfe's and other charities are well known. A sum now realising £5 17s. 1d. annually was left in 1713 for apprenticing one poor Deptford child per year, and the money is now distributed to the poor by the churchwardens, while some bequests, small in themselves, have been added to other and larger ones. A 1758 bequest realised now 11s. 10d. a-year, for "poor widows and householders who are communicants of the Church of England on 19th February." Another, dated 1783, was for "apprenticing the son of a bricklayer," and one, dated 1822, was for "buns and ale to the poor in the workhouse every Good Friday." Altogether nine charities have elapsed from various causes which in some cases could not be ascertained. The last accounts relative to the parochial charities showed total receipts to the amount of £156 2s. 8d., and an expenditure of £151 5s. 7d.

